# Visual Form Perception Can Be a Cognitive Correlate of Lower Level Math Categories for Teenagers

**DOI:** 10.3389/fpsyg.2017.01336

**Published:** 2017-08-04

**Authors:** Jiaxin Cui, Yiyun Zhang, Dazhi Cheng, Dawei Li, Xinlin Zhou

**Affiliations:** ^1^State Key Laboratory of Cognitive Neuroscience and Learning and IDG/McGovern Institute for Brain Research, Beijing Normal University Beijing, China; ^2^School of Psychology, Liaoning Normal University Dalian, China; ^3^Department of Pediatric Neurology, Capital Institute of Pediatrics Beijing, China; ^4^Center for Cognitive Neuroscience, Duke University Durham, NC, United States; ^5^Advanced Innovation Center for Future Education, Beijing Normal University Beijing, China

**Keywords:** visual form perception, approximate number system, numerical processing, mathematical achievement, computation

## Abstract

Numerous studies have assessed the cognitive correlates of performance in mathematics, but little research has been conducted to systematically examine the relations between visual perception as the starting point of visuospatial processing and typical mathematical performance. In the current study, we recruited 223 seventh graders to perform a visual form perception task (figure matching), numerosity comparison, digit comparison, exact computation, approximate computation, and curriculum-based mathematical achievement tests. Results showed that, after controlling for gender, age, and five general cognitive processes (choice reaction time, visual tracing, mental rotation, spatial working memory, and non-verbal matrices reasoning), visual form perception had unique contributions to numerosity comparison, digit comparison, and exact computation, but had no significant relation with approximate computation or curriculum-based mathematical achievement. These results suggest that visual form perception is an important independent cognitive correlate of lower level math categories, including the approximate number system, digit comparison, and exact computation.

## Introduction

Numerous behavioral studies have been conducted to assess the cognitive correlates of mathematical performance and have shown the important role of visuospatial processes in mathematical processing (e.g., Berg, 2008; Krajewski and Schneider, 2009; Simmons et al., 2012; Van Der Ven et al., 2013). Research in neuroscience also showed that visuospatial and mathematical processes recruit some common brain areas, such as the parietal cortex (see Hubbard et al., 2005, for a review). Most of the previous researches, however, were focused on the relationship between mathematical processing and visuospatial working memory (e.g., Berg, 2008; Krajewski and Schneider, 2009; Simmons et al., 2012; Van Der Ven et al., 2013; Sella et al., 2016). Although visuospatial working memory is a critical visuospatial component, it does not encompass all aspects of visuospatial processing. Indeed, visuospatial processing includes multiple components, such as visual perception, visual attention, spatial attention, and visuospatial working memory.

Visual perception, the starting point of visuospatial processing, had been shown to have a close relation with language processing through its relationship with orthographic processing in language processing (e.g., Damasio and Damasio, 1983; Eden et al., 1996; Demb et al., 1998; Cestnick and Coltheart, 1999; Talcott et al., 2000; Sperling et al., 2005; Meng et al., 2011; see Vidyasagar and Pammer, 2010, for a review). Given that mathematical processing is also based on symbolic systems (e.g., numbers, letters, mathematical signs, and even words) that are similar to symbolic languages, mathematical processing may also have a close relationship with visual perception. This relationship, however, had been only sparsely investigated.

### Visual perception and mathematical abilities

As mentioned above, visual perception may be related to not only language processing through its impact on orthographic processing, but also mathematical performance through its impact on the processing of mathematical symbols. However, only a few studies used visual perception tasks to show the relationship between visual perception and mathematical performance (e.g., Rosner, 1973; Solan, 1987; Kulp, 1999; Kurdek and Sinclair, 2001; Sigmundsson et al., 2010).

For example, Rosner (1973) measured visual perception with a visual perception test (VAT) (Rosner, 1969) in which the children were asked to copy designs drawn on dot matrices to match a target stimulus. The results showed that visual perception explained the variance in computation performance after controlling for auditory perception. A longitudinal study by Kurdek and Sinclair (2001) showed the importance of visual perception in children's math development. They used a visual discrimination task (perception and discrimination of similarities and differences between various shapes and geometric forms) as well as other tasks in a kindergarten readiness test (KRT) and found that visual discrimination ability in kindergarten children could uniquely predict mathematical achievement in fourth grade.

Parallel with the studies on the role of visual coherent motion detection in language performance, Sigmundsson et al.'s study 2011 on children with low mathematical performance found that the children were less sensitive to visual coherent motion than age-matched controls. Boets et al. (2011) further showed that coherent motion sensitivity predicted individual differences in simple subtractions. They reasoned that subtraction highly relies on quantity processing, which is subserved by regions along the intraparietal sulcus, a region in the visual dorsal pathway which underlies coherent motion detection (Boets et al., 2011).

### Visual form perception can account for the relation between the ANS and exact computation

The approximate number system (ANS) is shared by human and non-human animals. It functions by estimating the number of items (e.g., the number of dots in a dot array) without relying on counting one by one. The ANS acuity or accuracy has been shown to be closely associated with mathematical performance (e.g., Halberda et al., 2008; Libertus et al., 2011; Lyons and Beilock, 2011). For example, children with developmental dyscalculia also suffer from ANS impairment (e.g., Landerl et al., 2004; Geary et al., 2009; Piazza et al., 2010). Sensitivity of the ANS correlated with symbolic mathematical performance for normally developing children (e.g., Halberda et al., 2008, 2012; Mundy and Gilmore, 2009; Inglis et al., 2011; Libertus et al., 2011, 2013; Mazzocco et al., 2011; Bonny and Lourenco, 2013). Halberda et al. (2008) found that 14-year-old children's ANS ability was closely associated with their earlier symbolic mathematical performance in third grade, after controlling for a series of cognitive processes including general intelligence, language processing, and working memory.

Furthermore, ANS training (such as approximate arithmetic task) has been found to promote the development of symbolic computation abilities (e.g., Park and Brannon, 2013, 2014; Hyde et al., 2014), while a comment from Lindskog and Winman (2016) doubted the effect of Park and Brannon (2013, 2014) due to their methodology.

However, some studies failed to show the association (e.g., Sasanguie et al., 2012, 2013; Vanbinst et al., 2012; Zhou et al., 2015; see a review by de Smedt et al., 2013). Recently, Zhang et al. (2016) found that ANS acuity only correlated with mathematical fluency but not with mathematical reasoning.

The close relationship between the ANS and symbolic math performance was traditionally attributed to the domain-specific processing in the ANS (e.g., Barth et al., 2005, 2006, 2008; Nieder and Dehaene, 2009; Gilmore et al., 2010; Park and Brannon, 2013; Hyde et al., 2014). For example, Park and Brannon (2013) argued that humans' ANS permitted estimation and rough calculation of numerical quantities without symbols. Gilmore et al. (2010) treated ANS as a type of core numerical ability. The ANS is important for the acquisition of symbolic numerical skills, such as counting and arithmetic, because both ANS and symbolic numerical skills involve quantity processing (e.g., Gilmore et al., 2007; Inglis et al., 2011). Indeed, previous correlational studies have shown a significant relation between symbolic mathematics and quantity processing (e.g., de Smedt et al., 2009; Lefevre et al., 2010; Sasanguie et al., 2013; Zhang et al., 2016).

An alternative hypothesis was proposed by Zhou and colleagues (Zhou and Cheng, 2015; Zhou et al., 2015), who argued that visual form perception accounts for the close relation between the ANS and exact computation. Zhou et al. (2015) found that, after controlling for the score on geometric figure discrimination as well as the scores on other general cognitive processing measures (i.e., Raven's Progressive Matrices, mental rotation, choice reaction, visual tracing, and digit span), the close relation between the ANS and exact computation was not existent (e.g., Zhou et al., 2015). Zhou and Cheng (2015) also showed that the ANS difference between children with dyscalculia and typically-developed children was associated with difference in geometric figure discrimination. These results suggested a possibility that the ability of figure discrimination might account for the relation between the ANS and exact computation because the two previous studies have excluded some types of general cognitive factors (Zhou and Cheng, 2015; Zhou et al., 2015). That is, visual form perception is one of the possible reasons to interpret the positive relation between ANS and symbolic mathematical performance.

The geometric figure matching task used in the above two studies was adopted from the Manual for the Kit of Factor-Referenced Cognitive Tests (Ekstrom et al., 1976) and the visual perception test (VMI-VP) in the Beery-Buktenica Developmental Test of Visual-Motor Integration (VMI 4th edition, Beery et al., 1997). This task was used to measure the ability of geometric figure discrimination, which involves the abilities to attend to and to identify a figure's distinguishing features and details. The figure could be treated as a form or shape, consisting of abstract lines. The geometric figure matching task was used in the two studies because it could be associated with visual form perception, alphanumeric symbols, and numerosity.

Some previous studies found close relationships between the ANS and symbolic mathematical performance even after controlling for visual perception (Halberda et al., 2008). Halberda et al. (2008) controlled 4 types of visual processing tasks, including visual working memory, visual segmentation, object perception, and visual motor integration, and they still found significant relationships between the ANS and symbolic mathematical performance. Piazza et al. (2010) found that math education level did not relate to the precision of visual size discrimination in a size comparison task. Tibber et al. (2012) showed that the sensitivity to the orientations of two clusters of Gabor blobs could predict math abilities, but the sensitivity to size and density could not. The visual perception tasks used in these studies, however, were mostly focused on size, color, or brightness, which are different from visual form processing (e.g., Milner et al., 1991; Cavina-Pratesi et al., 2015). Thus, these tasks might not fully measure visual form processing. Given that visual form processing appears to be a critical component of visual perception with regard to relationships with the ANS and symbolic mathematics performance, tasks that are focused on visual form processing, such as the geometric figure discrimination task (Zhou and Cheng, 2015; Zhou et al., 2015) may be more proper to be utilized in studies of the relationships between visual form perception, the ANS, and symbolic mathematics performance.

### The current study

Although previous studies have shown the relation between visual perception and symbolic mathematical performance, they seldom differentiated between different categories of mathematical performance. Given that cognitive correlates for different types of mathematical performance differ greatly (e.g., Fuchs et al., 2008; Petrill et al., 2011), the role of visual perception might also differ among different types of mathematical performance. To address these two issues, the current study focused on the role of visual form perception, measured with the figure matching task which involves geometric figure discrimination (Zhou and Cheng, 2015; Zhou et al., 2015), in five typical categories of mathematical performance: numerosity comparison, digit comparison, exact computation, approximate computation, and curriculum-based math achievement.

According to previous studies on the relations between visual perception, ANS, and mathematical performance, it is hypothesized that visual perception is more closely associated with the performance in lower level math categories which rely more on visual features. The lower level math categories could include the ANS, digit comparison, and simple arithmetic. Previous studies have shown that visual form perception or the ANS was not significantly associated with higher level math categories, such as curriculum-based math achievement, which relies on math concept and math problem solving instead of lower level abilities (e.g., Sasanguie et al., 2012, 2013; Vanbinst et al., 2012; Zhou et al., 2015; Zhang et al., 2016). We thus predict that visual perception has minimal contribution to higher level math categories including approximate computation and curriculum-based math achievement.

Following the general hypothesis, we propose five specific hypotheses for the five math measures:

Hypothesis 1: visual form perception has a unique contribution to numerosity comparison. Numerosity comparison tasks usually use dot arrays to express quantity information. Patient studies showed that a patient suffering from visual form agnosia had deficit in numerosity processing (Milner et al., 1991). To extract numerical quantity information expressed by a dot array, the participants may transform the dot array into some type of visual form or pattern, which is related to visual form perception.
Hypothesis 2: visual form perception has a unique contribution to digit comparison. Digit comparison is based on visual perception of Arabic digits. The patient DF with visual form agnosia also had deficit in recognizing Arabic digits (Milner and Goodale, 2006).
Hypothesis 3: visual form perception has a unique contribution to exact computation. Studies have shown that visual form perception can account for the relation between ANS and exact computation (e.g., Zhou and Cheng, 2015; Zhou et al., 2015). Exact computation problems, such as 15–7 and 34 × 6, can be performed mentally and quickly. The symbolic processing is similar to geometric figure discrimination. Thus, visual form perception measured with geometric figure matching is expected to be associated with the exact computation.
Hypothesis 4: visual form perception has a minor or no unique contribution to approximate computation. Although the exact and approximate computation questions have similar presentation of Arabic digits, participants might just focus on some key digits (e.g., the most left digits) and neglect some digits to make quick approximation after having a glance at the whole presentation of approximate computation question. Approximate computation and curriculum-based math achievement could be typically associated with math problem solving (e.g., Jordan et al., 2009; Jitendra et al., 2014). Approximate computation (e.g., 645 × 54, 90.288 ÷ 22.8) could be categorized as a type of math problem solving, because the participants could not directly retrieve answers from long-term memory nor use routine procedures to calculate answers, but instead they have to search for solutions by flexibly applying strategies (Caviola et al., 2012; Ganor-Stern, 2015). Math problem solving involves the searching the path from preconditions to solutions and is supposed to include four steps: understanding the problem, devising a plan, carrying out the plan, and looking back (Polya, 1957). None of the steps appear to involve a large amount of visual form perception. Therefore, although visual form perception still affects recognition of math symbols (e.g., Arabic digits and mathematical signs), its importance is greatly reduced in approximate computation.
Hypothesis 5: visual form perception has a minor or no unique contribution to curriculum-based mathematical achievement. Curriculum-based math achievement typically involves math problem solving, such as simplifying algebraic expressions and solving equations and math word problems. In this study, the content in the mathematical achievement test was learnt during the past semester, in contrast to the exact computation test, whose content was learnt in lower grades of primary school (e.g., 1–3 grades). Therefore, the main processes in the mathematical achievement task could be the activation, retrieval, and application of math knowledge acquired during the past semester, which does not appear to heavily rely on visual form perception.

In addition, Hypothesis 5 is supported by a recent finding that, after controlling for some general cognitive processes (i.e., non-verbal IQ, mental rotation, visual tracing, verbal working memory, and choice reaction time), visual form perception has an independent contribution to exact computation, but not curriculum-based math achievement for third to fifth graders in primary schools (Zhou et al., 2015).

Several general cognitive processes, such as processing speed, attention, spatial working memory, and intelligence have been shown to be associated with mathematical performance (Berg, 2008; Krajewski and Schneider, 2009). To control for these general cognitive processes, these processes were measured and used as covariates in the current study.

## Methods

### Participants

The participants included 223 seventh grade students (90 boys and 133 girls, mean age 12.9 years-old (*SD* = 0.66), ranging from 11.2 to 13.7) in a middle school in Shijiazhuang municipality of Hebei Province in China. All participants were native Chinese speakers.

The school attended the study in order to assess the basic learning ability of its seventh graders and to perform remedial instruction partially based on the assessment. The program including the cognitive testing was fully explained to students' guardians (typically parents) in the school's parent meeting in the semester. During the meeting, the guardians gave written consent forms for the remedial instruction program. Among a total of 248 students, 223 students finally attended the cognitive testing. Testing results were provided only to the school's psychological counselors who possess psychological counseling certificates of the third or second level (highest level in China). No other teachers or administrators had access to the results. All tests were fully explained to the counselors, who were then able to use the results as evidence, along with the students' achievement scores and the teachers' subjective assessments to provide instructional suggestions for the students.

The school is public, with a slightly above-average academic level in the city. There were ~30–40 students per class. The study was approved by the institutional review board (IRB) at the State Key Laboratory of Cognitive Neuroscience and Learning at Beijing Normal University.

### Tests

A total of 11 tests were used. All except for the curriculum-based mathematical achievement test were computerized using web-based applications in the “Online Psychological Experimental System (OPES)” (www.dweipsy.com/lattice). Besides a figure matching test, which measured visual perceptual ability, other tests were also included to measure general cognitive processes, including choice reaction time, mental rotation, spatial working memory (adapted from Corsi Blocks Task, Corsi, 1972), visual tracing, and non-verbal matrices reasoning. The tests covered a wide range of cognitive processes that involve visual perception, attention, working memory and general intelligence. Previous studies have shown that these cognitive processes are closely associated with mathematical performance (e.g., Spinath et al., 2006; Deary et al., 2007; Berg, 2008; Krajewski and Schneider, 2009; Wei et al., 2012b).

**Figure 1:**
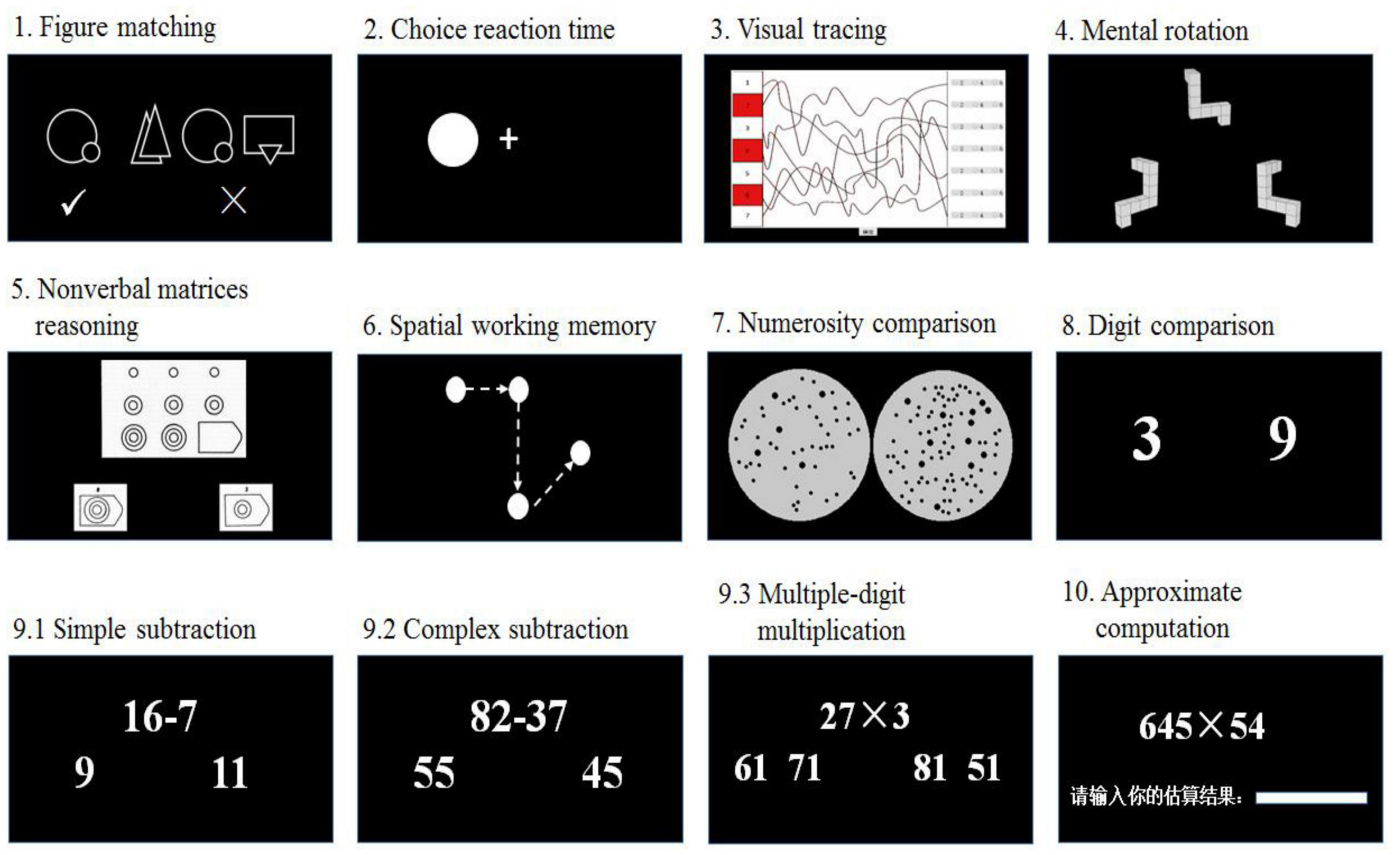
Schematic representation of 10 tests used in the study.

For most tests, the participants responded to two-choice options by pressing the Q and P keys on a computer keyboard to choose the correct answers. The other response modes were explained below in more details. In all tests except for the spatial working memory test, the participants were encouraged to respond as quickly and accurately as possible. The schematic representations of all tests, except for the curriculum-based mathematical achievement test, are displayed in Figure 1. The tests are introduced as follows.

#### Figure matching

The figure matching test was used to measure visual form perception ability (Zhou and Cheng, 2015; Zhou et al., 2015). The test included 120 trials, each containing one target line picture on the left side and three candidate line pictures on the right side. A picture consisted of two simple geometric figures. Previous studies have used geometric figure discrimination to measure visual form perception (e.g., Efron, 1969; Milner et al., 1991; Cavina-Pratesi et al., 2015). The four line pictures were presented simultaneously for 400 ms. Each picture had horizontal and vertical visual angles of 2.8°. The four pictures extended to a visual angle of about 15°. The participants were asked to fixate at the center of the screen in the beginning of the experiment, but no fixation sign was presented for each trial. The participants were asked to judge whether the picture on the left side was the same as any of the pictures on the right side. The 120 trials were grouped into three 40-trial sessions, and the participants were asked to complete all trials. There were 60 matched trials and 60 non-matched trials. The trials were constructed from 150 line figures, each picture being used 1–3 times.

The score of this test was calculated as the adjusted number of correct trials to control for the effect of guessing in multiple choice tests. The score was calculated by subtracting the number of incorrect responses from the number of correct responses (e.g., Salthouse, 1994; Salthouse and Meinz, 1995; Hedden and Yoon, 2006; Cirino, 2011). This procedure followed the Guilford correction formula “*S* = *R-W/ (n-1)*” (*S*: the adjusted number of items that the participants can actually perform without the aid of chance. *R*: the number of right responses. *W*: the number of wrong responses. *n*: the number of alternative responses to each item) (Guilford and Guilford, 1936). This correction procedure has been utilized recently in studies of mathematical cognition (e.g., Cirino, 2011; Wei et al., 2012a,b) and cognition in general (e.g., Salthouse, 1994; Putz et al., 2004; Hedden and Yoon, 2006).

The same scoring procedure was used for the other tests, if not specified otherwise below.

#### Choice reaction time

A basic reaction time task was employed to control for effects of manual response and mental processing speed (cf., Butterworth's (2003) “Dyscalculia Screener”, which included a reaction time task). Each trial consisted of a fixation cross in the center of the computer screen and a white dot either to the left or right of the fixation cross. The participants responded to the dot on the left side with the left hand and to the dot on the right side with the right hand. This test included 30 trials. The RSI (response-stimulus interval) was varied randomly between 1,500 and 3,000 ms.

The score of this test was calculated as the median reaction time and error rate for each participant. The gross mean error rate for the choice reaction time task was very low (1.3%) and was not further analyzed.

#### Visual tracing

The test was adapted from Groffman's visual tracing test (Groffman, 1966) and was used to assess the ability of oculomotor coordination, which has been linked to reading disability (Groffman, 1994) and mathematical deficit (Fischer et al., 2008; Groffman, 2009). Several interweaving curved lines were presented within a square, starting from the left side of the square and ending on the right side. The participants were asked to track a particular line from the beginning to the end only by “eyeing” (i.e., they were not allowed to use a finger, the cursor, or an object to trace) and then to mark the correct end point by clicking the left mouse button. This task became increasingly difficult as the total number of lines increased. There were 12 groups of trials, each containing three trials. In each group of trials, the target curved lines were interweaved with each other and also interweaved with other non-target curved lines. The participants were instructed to mark the end points of the target lines with red color. This task contained 181 trials and was limited to 4 min.

The participants earned one score if they correctly clicked on the end point of a target curved line. The final score of this test was the sum of scores.

#### Mental rotation

Mental rotation has been treated as a representative of active visuospatial working memory, because it involves active manipulation of spatial information, other than just passive memorizing and retrieval (Vecchi and Girelli, 1998). The mental rotation test was adapted from Vandenberg and Kuse (1978). The revised version had only two choices and had a 3 min limit for completion. Each trial consisted of three three-dimensional images. One target image was presented on the top of the screen. The participants mentally rotated the target image to match one of two candidate images that were presented at the bottom of the screen. One candidate image was rotated from the target image with a rotation angle ranging from 15° to 345° (with intervals of 15°). The other image was a mirror image of the target. In each trial, the stimuli remained on the screen until the participant responded.

#### Nonverbal matrix reasoning

Nonverbal matrix reasoning was utilized to assess the abstract reasoning ability. Previous research has shown that nonverbal matrix reasoning score correlated with mathematical performance (Rohde and Thompson, 2007; Kyttälä and Lehto, 2008). The task was adapted from Raven's Progressive Matrices (Raven, 2000). The participants were instructed to figure out hidden rules underlying a presented figure and to select the missing segment of the figure from several candidate answers. We used a simplified version of the test, which included only two candidate answers for each question, instead of 4~6 options in the original test. Thus, the number of candidate answers was consistent with the other tests in this study. Due to limited time, the test was shortened to 80 items including 44 items from the Standard Progressive Matrices (12 items from the first set and eight items from each of the other four sets) and 36 items from the Advanced Progressive Matrices. The formal test was limited to 4 min. Similar shortened versions of this test have been used in previous studies (e.g., Bouma et al., 1996; Bors and Vigneau, 2001; Vigneau et al., 2006; Wei et al., 2012a). The shortened version had convergent validity, as shown by its high correlation with a number series completion task, which measures a type of reasoning in mathematics (Wei et al., 2012a). According to previous studies (Chuderski, 2013, 2015), this test with limited time would be related to working memory.

#### Spatial working memory

This test was adapted from the Corsi Blocks Task (Corsi, 1972). Dots were sequentially presented in an implicit 3 × 3 lattice on the computer screen. The number of dots for a trial ranged from three to nine. Each dot was presented for 1,000 ms, with an interval of 1,000 ms between dots. After the presentation of all dots, the participants used a mouse to click on the lattice according to the position and order of the dot presentation.

The score of this test was calculated as accuracy, using the following formula: Accuracy = 100 – | response – standard answer | / (standard answer + | response – standard answer |) × 100. The formula returns values from 0 to 100. *Response* refers to the participants' answer, and *standard answer* refers to the correct answer. Deviation of the participants' answer from the standard answer was divided by the sum of the standard answer and the deviation, which gives the degree of deviation from the standard value. For each trial, two accuracy scores were first calculated. The accuracy scores along the horizontal and vertical directions were calculated using the x and y coordinates, respectively, of the target dot and the response location. A final accuracy score was then calculated as the average of these two scores. For example, the coordinates of a target dot is (10, 10), and those of the response is (12, 15). The accuracy of x coordinate is 100-|(12-10)|/ (10+|(12-10)|)×100=83.33%, and the accuracy of y coordinate is 100-|(15-10)|/ (10+|(15-10)|)×100=66.67%. The average accuracy for the target dot is 75.00%.

The formula was adapted from the formula for the percentage absolute error (PAE) in the number-line task (Siegler and Mu, 2008): PAE = |estimate – estimated quantity | /scale of estimates. Given that the participants could provide any number as the solution in some cases (e.g., approximate computation task introduced below), there is no limit for the participants' response. To address this issue, we revised the denominator in the Siegler and Mu's formula. The formula is typically used to calculate the deviation of response from standard answer. Thus, “standard answer” was treated as the reference and the denominator. Meanwhile, the absolute difference “| response—standard answer |” was also added to the denominator to ensure that the ratio in the new formula was in the range between 0 to 1 (inclusively), for any number in both the response and the standard answer.

#### Numerosity comparison

The numerosity comparison test was used to assess approximate number sense (Wei et al., 2012a). Each trial consisted of two dot arrays presented for 200 ms, the same as in Halberda et al.'s study study (2008), which tested 14-year-old children. Each dot array included 11, 14, 17, 20, 23, 26, or 29 dots. The visual angle for a dot array presented in a gray circle is 6.8°. The diameters of dots varied from 1 to 7 mm. The two dot arrays in a trial were horizontally aligned and extended to a visual angle of about 14°. The participants were asked to fixate at the center of the screen in the beginning of the task, but no fixation sign was presented for each trial. The participants were instructed to choose the dot array with more dots, ignoring all visual properties, including total surface area, envelope area, diameter, and circumference. The test includes 120 trials. The dot arrays were created following a common procedure to control for continuous quantities in non-symbolic numerical discrimination (Halberda et al., 2008; Agrillo et al., 2013). The two dot arrays in half of the trials had the same total combined area, whereas those in the other half had the same average area of all dots. The dots in a dot array were randomly distributed within a circle, and the dots' sizes varied. The envelope area/convex hull varied little from trial to trial. There were 6 ratio types between the numbers of dots in the two dot arrays, ranged from 1.2 to 2.0. There were 20 trials from each ratio type. The trials were administered in three sessions, with 40 trials for each session. The students were asked to complete all of the trials.

Gebuis and Reynvoet (2011) proposed that five visual properties of the numerosity comparison task could affect numerosity comparison: total surface area, envelope area or convex hull, item size, density, and circumference. Density is defined as the number of items per unit area (e.g., Anobile et al., 2013a; Tinelli et al., 2015). Zhou et al. (2015) showed that the numerosity comparison task was still ratio-dependent after the five visual properties of dot arrays were controlled for. To confirm previous findings, the current study would also examine the effects of visual properties on task performance.

#### Digit comparison

The digit comparison test was used to measure the ability of symbolic numerical comparison and it included 84 pairs of single-digit Arabic numbers (ranging from 2 to 9) presented in random orders. The participants were asked to choose the number that was larger in quantity. The formal test was limited to 2.5 min.

#### Exact computation

There were three exact computation tasks: simple subtraction, complex subtraction, and multiple-digit multiplication. Scores for the three tasks were averaged as the score of exact computation.

##### Simple subtraction

The simple subtraction task, consisting of 92 problems, was the reversed operation to single-digit addition. The largest minuend was 18, and the smallest one was 2. The differences between two operands were always single-digit numbers. Each problem included two candidate answers. The false answer deviated from the true answer by plus or minus 3 (i.e., ±1, ±2, or ±3). This was a time-limited (2 min) task.

##### Complex subtraction

The complex subtraction task, consisting of 95 problems, included double-digit numbers for both operands. Borrowing was required for most problems. Each problem included two candidate answers. The differences between the false answers and the true answers were 1 or 10. The task was limited to 2 min.

##### Multiple-digit multiplication

The multiple-digit multiplication task consisted of 76 problems and included a double-digit number multiplied by a single-digit number (e.g., 27 × 3). Carrying was required for each problem. Each problem included four candidate answers, one being the true answer and the other three being false answers. The differences between the true answers and the false answers were times of 10, ranging from 10 to 90. The four candidate answers were divided into two groups, with two answers in a group. The two groups were presented on the left and right sides, respectively, of the screen below the question. The participants were asked to judge on which side the correct answer was presented. The task was limited to 2 min.

#### Approximate computation

During the approximate computation test, the participants estimated the results of computation problems that were typically too complex to be solved mentally using exact computation within the given time interval. In each trial, a computation problem was presented on the screen, which involved addition, subtraction, multiplication, or division (e.g., 4578 + 8144, 208.3-179.04, 645 × 54, 90.288 ÷ 22.8). The participants were instructed to estimate the results of the computation problems and then fill the result in a dialog box on the bottom of the screen using the keyboard. Each trial should be finished within 15 s, otherwise a participant would receive a score 0. There were 40 trials, and each participant was required to finish all trials.

The scoring method for the approximate computation task was the same as that for spatial working memory task. For example, 76 × 88, as a trial of this task, has a standard answer of 6688. If a participant gave his answer as 6600, then his score of this trial is 100-|(6600-6688)|/ (6688+|(6600-6688)|)×100=98.70%.

#### Curriculum-based math achievement

Besides these cognitive tests, general achievement scores for mathematics in the final examination of the semester were obtained from the school. The math achievement score was treated as a measure of general mathematical performance. The achievement test was developed by the Instruction Research Unit, affiliated to the local Department of Education, and administered to all students in the district at the end of each semester. The test was curriculum-based and covered computation, mathematical concepts, and applied problem solving. Students had 90 min to complete each test. The current study used the scores from the tests provided by the middle school that participated in the study.

### Procedure

The battery of tests was administered in two 40-min sessions. The practice session included four to six trials, which were similar to those used in the formal testing. The feedback for correct answers for all cognitive tasks was “Correct! Can you go faster?,” and that for incorrect answers was “It is wrong. Try again.” During the practice session, the children could ask the experimenters any questions related to the testing. The formal testing began after the children had finished the practice session.

Computerized tests were administered to the students (one class at a time) in a computer classroom. Each class was monitored by two experimenters as well as the teacher of that class. The experimenters explained the instruction with slides for each task. The teacher was present only for the purpose of discipline (e.g., maintaining silence during the formal testing). After all the students completed one test, the experimenter started to administer the next test. For each test, the students were first given instructions and then completed a practice session, followed by the formal testing. Only when all students understood the procedure in practice, could they begin the formal testing. After the main experimenter said “Start!,” all children in the computer classroom pressed a key to begin the formal testing. All students performed the tasks in the same order.

The data collection took place between March and June, 2014.

### Data analysis

First, descriptive statistics was performed for all tests. Mean, standard deviation and split-half reliability were calculated for each test. Spearman-Brown formula was used to adjust for the test length when calculating split-half reliabilities. Pearson's correlation coefficients were calculated between the scores of all cognitive and mathematical tests.

Second, a series of hierarchical regression analyses were conducted to test the role of visual form perception in the five categories of mathematical performances, while controlling for age, gender, and all other 5 types of general cognitive processes.

Third, a path model was used to test the structural relationships among all measures in the current study. The path analysis was performed in the IBM SPSS Amos 20 software, using maximum likelihood estimation. The chi-square, root mean square error of approximation(RMSEA), comparative fit index (CFI) and standard root mean square residual (SRMR) were used to assess model fit. According to Kline (2005), good model fit was indicated by a non-significant chi-square, RMSEA values less than 0.05, CFI values greater than 0.95, and SRMR values less than 0.10. The hypothesized path model assumes that the five general cognitive processes (i.e., choice reaction time, visual tracing, mental rotation, spatial working memory, and non-verbal matrix reasoning) contribute to the five categories of mathematical performance, and that figure matching only contributes to numerosity comparison, digit comparison, and exact computation. The above model was compared with a full-connection model, which added two additional links (figure matching and approximate computation, and figure matching and curriculum-based math achievement).

Fourth, mediation analyses were conducted with the bootstrapping method (Preacher and Hayes, 2008) to investigate the contribution of visual form perception in mediating the relation between numerosity comparison and exact computation, after controlling for the general cognitive processing variables (choice reaction time, visual tracing, mental rotation, spatial working memory, and non-verbal matrix reasoning), as well as age and gender.

Last, a series of Bayesian regression analyses using Matlab were conducted to test the two null hypotheses (4 and 5), to test Bayesian estimation relying on the probability beyond linear regressions. According to Kleiman et al. (2014), Beta weights and 95% confidence intervals (also called credibility or probability intervals) were produced, and the 95% confidence intervals without including zero were considered as significance at *p* < 0.05.

## Results

The reaction times of the correct trials for each participant were trimmed for the choice reaction time test. That is, we deleted the correct trials with reaction times out of the mean ± 3 standard deviations. 2.15% trials were deleted accordingly.

### Descriptive statistics

Means and standard deviations of scores for all the general cognitive processing and the 5 categories of mathematical performance are displayed in Table 1. The split-half reliability for each cognitive test is also shown in Table 1. All tests have acceptable reliabilities, ranging from 0.84 to 0.96.

**Table 1:** Descriptive statistic and split-half reliability for all the tasks used for middle school students.

**Task**	**Index**	**Mean (SD)**	**Split-half reliability**
Figure matching	Proportion correct (%)	71.70 (10.56)	0.88
Choice reaction time	Reaction time (ms)	336.48 (81.18)	0.96
Visual tracing	No. of correct trials	19.23 (5.14)	0.92
Mental rotation	Adjust no. of correct trials	20.45 (9.49)	0.87
Nonverbal matrix reasoning	Adjust no. of correct trials	20.48 (7.51)	0.84
Spatial working memory	Accuracy (%)	82.58 (4.73)	0.92
Numerosity comparison	Proportion correct (%)	77.58 (8.82)	0.91
Digit comparison	Adjust no. of correct trials	76.11 (16.49)	0.88
Exact computation	Adjust no. of correct trials	94.49 (19.52)	0.86
Approximate computation	Accuracy (%)	69.49 (12.52)	0.90
Curriculum-based math achievement	Score(0–100)	81.25 (17.94)	

*Adjust no. of correct trials = Total correct trials minus total incorrect trials. This adjustment was made to control for the effect of guessing in multiple choice tests. Four tests used this adjustment. Without the adjustment, the accuracy and reaction time for the tests are as following: 90.80% and 542 ms for digit comparison, 87.91% and 1,944 ms for exact computation, 74.07% and 3,284 ms for nonverbal matrix reasoning, and 76.85% and 3,551 ms for mental rotation*.

Performance in the numerosity comparison task was first examined with a partial correlation analysis to ensure that the participants performed the task in a ratio-dependent manner. After controlling for the five visual properties (i.e., total surface area, envelope area or convex hull, item size, density, and circumference, see Gebuis and Reynvoet, 2011), accuracy across all trials was still ratio-dependent, *r*_(113)_ = 0.41, *p* < 0.001.

Pearson's correlation coefficients among all measures with Bonferroni correction are displayed in Table 2. A Bonferroni correction was used to maintain the *p*-value < 0.05 across the 78 correlations in Table 2. Thus, a conservative *p*-value of < 0.00064 (=0.05/78) was considered statistically significant.

**Table 2:** Intercorrelations among all cognitive processing scores based on Pearson's correlation.

	**1**	**2**	**3**	**4**	**5**	**6**	**7**	**8**	**9**	**10**	**11**	**12**
1. Figure matching	–											
2. Choice reaction time	−0.18	–										
3. Visual tracing	0.24^*^	−0.25^*^	–									
4. Mental rotation	0.31^*^	−0.12	0.30^*^	–								
5. Nonverbal matrix reasoning	0.24^*^	−0.24^*^	0.27^*^	0.30^*^	–							
6. Spatial working memory	0.22^*^	−0.23^*^	0.28^*^	0.31^*^	0.22^*^	–						
7. Numerosity comparison	0.33^*^	−0.07	0.06	0.22^*^	0.22^*^	0.16	–					
8. Digit comparison	0.32^*^	−0.06	0.13	0.16	0.23^*^	0.25^*^	0.28^*^	–				
9. Exact computation	0.44^*^	−0.09	0.21	0.13	0.26^*^	0.26^*^	0.23^*^	0.49^*^	–			
10. Approximate computation	0.19	−0.14	0.24^*^	0.21	0.23^*^	0.25^*^	0.23^*^	0.39^*^	0.38^*^	–		
11. Curriculum-based math achievement	0.27^*^	−0.21	0.31^*^	0.27^*^	0.36^*^	0.21^*^	0.16	0.34^*^	0.51^*^	0.48^*^		
12. Age	−0.09	0.09	−0.18	−0.01	−0.08	0.02	0.03	−0.05	−0.02	−0.09	−0.08	
13. Gender	−0.02	0.05	−0.03	−0.13	0.08	0.01	−0.13	0.15	0.24^*^	0.05	0.19	−0.02

* *p < 0.05, with Bonferroni correction for multiple tests. The gender variable was coded as 1 (for boys) and 2 (for girls)*.

### Linear hierarchical regression analyses

Linear hierarchical regression analyses for numerosity comparison, digit comparison, exact computation, approximate computation, and curriculum-based mathematical achievement were conducted separately to test the 5 hypotheses. Tables 3–**7** show the results. According to Tables 3–**7**, visual form perception had unique contributions to numerosity comparison, digit comparison, and exact computation, but had no significant contribution to approximate computation and had only a minor contribution to curriculum-based math achievement. These results are presented in the following five sections. Thus, subsections in Section Results include five hierarchical regression analyses, as shown in Tables 3–**7**.

**Table 3:** Results from hierarchical multiple regression analysis for the relations of figure matching and numerosity comparison.

**Predictors**	**Step 1 β**	**Step 2 β**	**Step 3 β**
Age	0.03	0.03	0.05
Gender	−0.13	−0.13	−0.13
Choice reaction time	–	−0.00	0.02
Visual tracing	–	−0.06	−0.08
Mental rotation	–	0.14	0.08
Nonverbal matrix reasoning	–	0.18	0.15
Spatial working memory	–	0.09	0.06
Figure matching	–	–	0.28^*^
	*R*^2^ = 0.02	*R*^2^ = 0.10	*R*^2^ = 0.17
		(Δ*R*^2^ = 0.08^*^)	(Δ*R*^2^ = 0.07^*^)

* *p < 0.05, with Bonferroni correction*.

We also performed Bonferroni correction on the 5 regression analyses. Thus, a conservative *p*-value of <0.01 (=0.05/5) was considered statistically significant.

#### Visual form perception and numerosity comparison

According to Table 3, general cognitive processing excluding visual form perception could account for 8.1% variation of numerosity comparison, *F*_*change*__(5, 215)_ = 3.86, *p* = 0.002. After controlling for scores of the five general cognitive processing (choice reaction time, visual tracing, mental rotation, spatial working memory, and non-verbal matrix reasoning) as well as gender and age, figure matching still accounted for 6.6% variance of numerosity comparison, *F*_*change*__(1, 214)_ = 16.94, *p* < 0.001. The result is as expected by Hypothesis 1.

#### Visual form perception and digit comparison

According to Table 4, general cognitive processing except for visual form perception accounted for 9.4% variations of digit comparison, *F*_*change*__(5, 215)_ = 4.58, *p* = 0.001. Visual form perception accounted for 5.8% of variance of digit comparison after controlling for the five general cognitive processing as well as gender and age, *F*_*change*__(1, 214)_ = 15.16, *p* < 0.001. The result is as expected by Hypothesis 2.

**Table 4:** Results from hierarchical multiple regression analysis for the relations of figure matching and digit comparison.

**Predictors**	**Step 1 β**	**Step 2 β**	**Step 3 β**
Age	−0.05	−0.04	−0.03
Gender	0.15	0.15	0.15
Choice reaction time	–	0.03	0.06
Visual tracing	–	0.01	−0.01
Mental rotation	–	0.08	0.02
Nonverbal matrix reasoning	–	0.15	0.12
Spatial working memory	–	0.20^*^	0.17
Figure matching	–	–	0.26^*^
	*R*^2^ = 0.03	*R*^2^ = 0.12	*R*^2^ = 0.18
		(Δ*R*^2^ = 0.09^*^)	(Δ*R*^2^ = 0.06^*^)

* *p < 0.05, with Bonferroni correction*.

#### Visual form perception and exact computation

Table 5 shows the regression results of exact computation on general cognitive processing as well as gender and age. The general cognitive processing, excluding visual form perception, accounted for 11.2% variation in exact computation, *F*_*change*__(5, 215)_ = 5.82, *p* < 0.001. Visual form perception accounted for another 13.2% in exact computation after the 5 general cognitive processing were controlled, *F*_*change*__(1, 214)_ = 40.30, *p* < 0.001. The result is as expected by Hypothesis 3.

**Table 5:** Results from hierarchical multiple regression analysis for the relations of figure matching and exact computation.

**Predictors**	**Step 1 β**	**Step 2 β**	**Step 3 β**
Age	−0.02	0.02	0.04
Gender	0.24^*^	0.23^*^	0.23^*^
Choice reaction time	–	0.01	0.04
Visual tracing	–	0.12	0.08
Mental rotation	–	0.02	−0.06
Nonverbal matrix reasoning	–	0.16	0.12
Spatial working memory	–	0.18^*^	0.15
Figure matching	–	–	0.39^*^
	*R*^2^ = 0.06^*^	*R*^2^ = 0.17	*R*^2^ = 0.30
		(Δ*R*^2^ = 0.11^*^)	(Δ*R*^2^ = 0.13^*^)

* *p < 0.05, with Bonferroni correction*.

#### Visual form perception and approximate computation

Table 6 showed the regression results of approximate computation on general cognitive processing as well as gender and age. General cognitive processing excluding visual form perception accounted for 11.4% variation in approximate computation, *F*_*change*__(5, 215)_ = 5.63, *p* < 0.001, similar to their contribution to exact computation. However, visual form perception did not have an additional contribution to approximate computation, Δ*R*^2^ = 0.005, *F*_*change*__(1, 214)_ = 1.19, *p* = 0.276. The result is as expected by Hypothesis 4.

**Table 6:** Results from hierarchical multiple regression analysis for the relations of figure matching and approximate computation.

**Predictors**	**Step 1 β**	**Step 2 β**	**Step 3 β**
Age	−0.09	−0.06	−0.06
Gender	0.05	0.06	0.06
Choice reaction time	–	−0.03	−0.02
Visual tracing	–	0.12	0.12
Mental rotation	–	0.09	0.08
Nonverbal matrix reasoning	–	0.12	0.11
Spatial working memory	–	0.15	0.14
Figure matching	–	–	0.08
	*R*^2^ = 0.01	*R*^2^ = 0.13	*R*^2^ = 0.13
		(Δ*R*^2^ = 0.11^*^)	(Δ*R*^2^ = 0.01)

* *p < 0.05, with Bonferroni correction*.

#### Visual form perception and curriculum-based math achievement

Table 7 showed the regression results of curriculum-based math achievement on general cognitive processing as well as gender and age. General cognitive processing excluding visual form perception accounted for 20.2% variance in math achievement, *F*_*change*__(5, 215)_ = 11.49, *p* < 0.001. Visual form perception could not show any significant contribution to mathematical achievement. Δ*R*^2^ = 0.014, *F*_*change*__(1, 214)_ = 4.02, *p* = 0.552. The result is as expected by Hypothesis 5.

**Table 7:** Results from hierarchical multiple regression analysis for the relations of figure matching and curriculum-based math achievement.

**Predictors**	**Step 1 β**	**Step 2 β**	**Step 3 β**
Age	−0.08	−0.02	−0.01
Gender	0.19^*^	0.20^*^	0.20^*^
Choice reaction time	–	−0.09	−0.08
Visual tracing	–	0.17	0.16
Mental rotation	–	0.15	0.12
Nonverbal matrix reasoning	–	0.22^*^	0.21^*^
Spatial working memory	–	0.05	0.04
Figure matching	–	–	0.13
	*R*^2^ = 0.04	*R*^2^ = 0.25	*R*^2^ = 0.26
		(Δ*R*^2^ = 0.20^*^)	(Δ*R*^2^ = 0.01)

* *p < 0.05, with Bonferroni correction*.

### Bayesian regression analyses

The above linear hierarchical regression analyses have shown that visual perception might have significant linear correlation to numerosity comparison, digit comparison, and exact computation, but not to approximate computation and curriculum-based mathematics achievement, after controlling for other types of general cognitive processes. In order to test the two null hypotheses (4 and 5), we used Bayesian regression analyses to check whether visual perception would have some non-linear relationship with approximate computation and curriculum-based mathematics achievement. The results were shown in Tables 8, 9, using the same steps as those in Tables 3–7.

**Table 8:** The parameter estimates for the repeated-measures Bayesian model on the relations between figure matching and approximate computation (using the same steps as those in Table 6).

**Predictors**	**B (95% CI)**	***p***	***R*^2^ (95% CI)**
Step 1			0.011 (0.000–0.047)
Age	−0.15 (−0.36–0.06)	0.915	
Gender	1.33 (-2.04–4.69)	0.219	
Step 2			0.126 (0.045–0.266)
Choice reaction time	−0.00 (-0.03–0.02)	0.664	
Visual tracing	0.30 (-0.04–0.64)	0.042	
Mental rotation	0.12 (-0.07–0.31)	0.102	
Nonverbal matrix reasoning	0.20 (−0.03–0.43)	0.045	
Spatial working memory	0.40 (0.03–0.76)	0.016	
Step 3			0.131 (0.049–0.270)
Figure matching	0.04 (−0.03–0.10)	0.138	

**Table 9:** The parameter estimates for the repeated-measures Bayesian model on the relations between figure matching and curriculum-based math achievement (using the same steps as those in Table 7).

**Predictors**	**B (95% CI)**	***p***	***R*^2^ (95% CI)**
Step 1			0.043 (0.006–0.111)
Age	−0.18 (−0.47–0.12)	0.879	
Gender	6.93 (2.18–11.67)	0.002	
Step 2			0.245 (0.131–0.415)
Choice reaction time	−0.02 (−0.05–0.01)	0.918	
Visual tracing	0.60 (0.14–1.06)	0.005	
Mental rotation	0.28 (0.04–0.54)	0.012	
Nonverbal matrix reasoning	0.53 (0.22–0.84)	0.000	
Spatial working memory	0.19 (−0.30–0.67)	0.226	
Step 3			0.258 (0.147–0.427)
Figure matching	0.09 (0.00–0.18)	0.023	

Table 8 showed the results of the Bayesian regression analysis for Hypothesis 4. Only general cognitive processing abilities excluding visual form perception were significant predictors for approximate computation. Visual form perception also had no additional contribution to approximate computation (Δ*R*^2^ = 0.005), similar to the result from the above linear regression analysis.

Table 9 showed the results of the Bayesian regression analysis for Hypothesis 5. Only general cognitive processing abilities excluding visual form perception were significant predictors for curriculum-based math achievement. Visual form perception also had no additional contribution to curriculum-based math achievement (Δ*R*^2^ = 0.013), similar to the result from the above linear regression analysis.

### Path model analysis and mediation analyses

**Figure 2:**
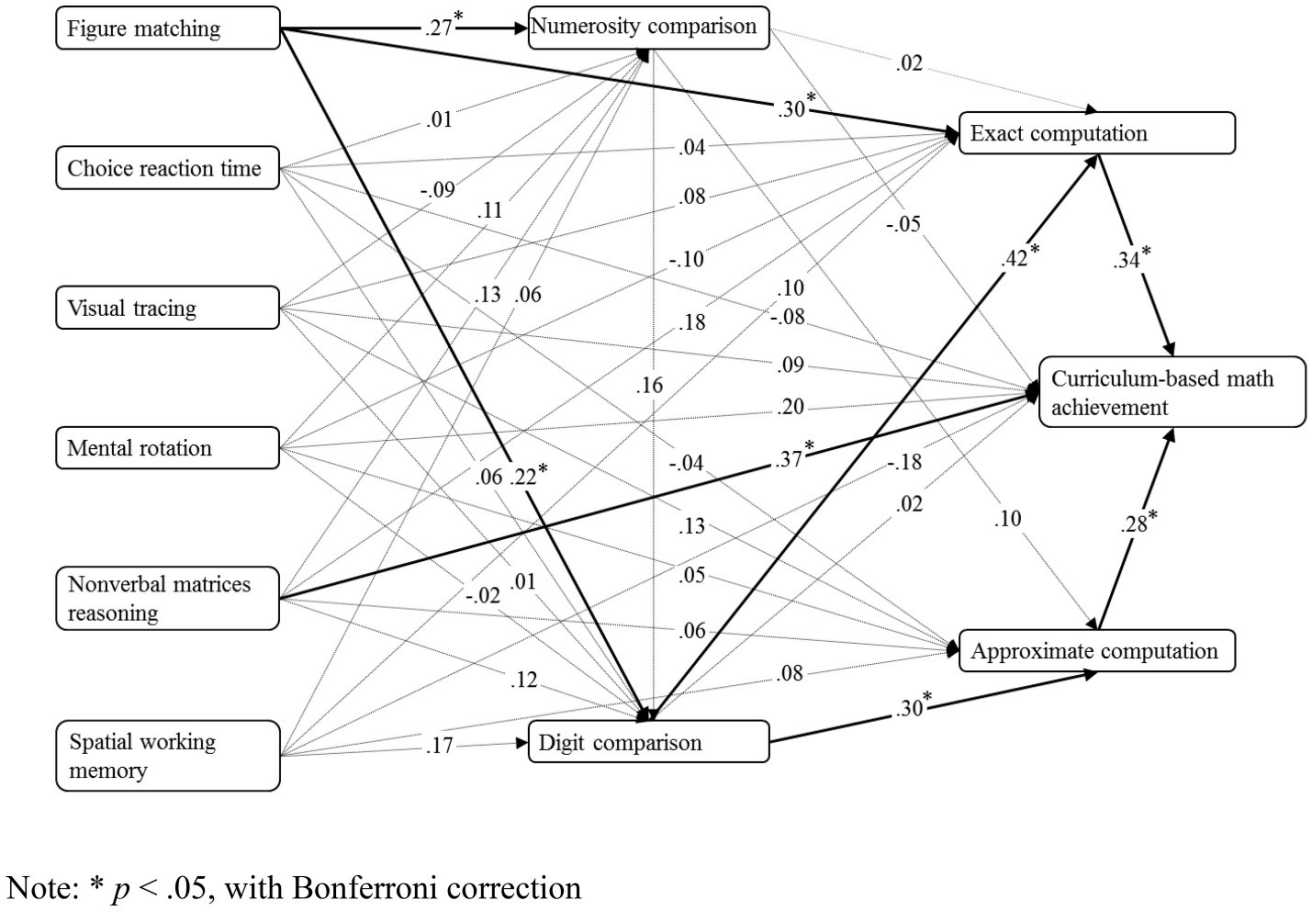
Path model for six general cognitive processes and five categories of mathematical performance (factor loadings are standardized, *N* = 223). ^*^p < 0.05, with Bonferroni correction.

Figure 2 shows the path model for the structural relationships among all six types of cognitive abilities and five types of mathematical performances, and for the contribution of lower level of mathematical performance to higher level of mathematical performance. The hypothesized model was a good fit for the data, $\chi_{\left(3\right)}^{2}$ = 3.73, *p* = 0.292, RMSEA = 0.03, CFI = 1.00, and SRMR = 0.02. The model showed that only visual form perception in all six types of cognitive abilities had significant direct associations with numerosity comparison, digit comparison, and exact computation. Numerosity comparison did not display any significant relation with the other four categories of mathematical performance in the path model. All the significance of loading in the path model was under Bonferroni correction. A Bonferroni correction was used to maintain the *p*-value < 0.05 across the 35 links in Figure 2. Thus, a conservative *p*-value of < 0.0014 (=0.05/35) was considered statistically significant.

After removing all the non-significant relations in Figure 2, the new model was also a good fit for the data, $\chi_{\left(5\right)}^{2}$ = 2.27, *p* = 0.881, RMSEA = 0.00, CFI = 1.00, and SRMR = 0.02.

The full-connection model with two additional links (figure matching and approximate computation, and figure matching and curriculum-based math achievement) was not a good fit for the data, $\chi_{\left(2\right)}^{2}$ = 12.78, *p* = 0.002, RMSEA = 0.16, CFI = 0.98, and SRMA = 0.28.

**Figure 3:**
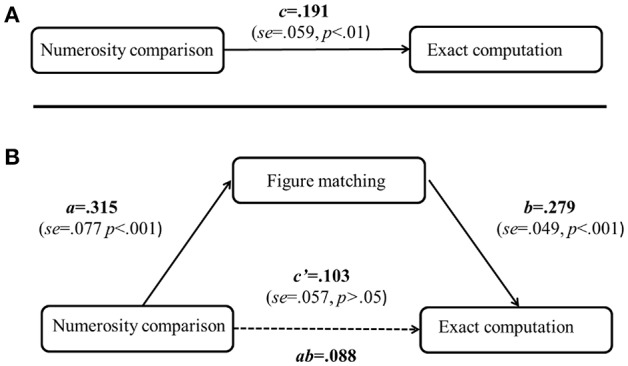
Mediation analyses for the contributions of numerosity comparison to exact computation. The top panel **(A)** is for the direct effect of numerosity comparison on exact computation, the bottom panel **(B)** is for the mediation effect of figure matching on the relation between numerosity comparison and exact computation. For the mediation analysis, general cognitive processing (choice reaction time, visual tracing, mental rotation, spatial working memory, and non-verbal matrix reasoning) as well as age and gender differences have taken as controlled covariates. The model is constrained by the assumption of c = ab + c′. c: direct effect of the original predictor; ab: indirect effect of the mediator, and c': the remaining (unmediated) direct effect.

For the mediation analyses, the dependent variable was the exact computation, and the controlled covariates were the general cognitive processing variables (choice reaction time, visual tracing, mental rotation, spatial working memory, and non-verbal matrix reasoning), as well as age and gender. We tested whether visual form perception mediated the relation between numerosity comparison and exact computation (see Figure 3A). Results showed that the relation between numersoity comparison and exact computation was fully mediated by visual form perception (see Figure 3B).

## Discussion

The current study aimed at investigating the roles of visual form perception in five categories of mathematical performance. A total of 223 students in the first grade of a middle school performed a series of cognitive and mathematical tasks. Hierarchical regression analyses showed that visual form perception had substantial contributions only to numerosity comparison, digit comparison, and exact computation, but had no substantial contribution to approximate computation or curriculum-based mathematical achievement after controlling for gender, age, and scores of all other available measures of general cognitive processes including choice reaction time, visual tracing, mental rotation, non-verbal matrix reasoning, and spatial working memory. This pattern was unique to visual form perception and wasn't replicated in other cognitive processes. Path model that further validated the results can be found in the hierarchical regression analyses above.

### Roles of visual form perception in numerical quantity comparison

Visual form perception had independent contribution to the ANS (i.e., numerosity comparison) after the other general cognitive processes (i.e., choice reaction time, visual tracing, mental rotation, non-verbal matrix reasoning, and spatial working memory) were controlled for (Table 3). The close relation between visual form perception and the ANS is consistent with results found in previous studies (e.g., Burr and Ross, 2008; Tibber et al., 2012; Anobile et al., 2013b).

Although some behavioral studies have investigated the relationships between visual perception and mathematical performance (e.g., Rosner, 1973; Rourke and Finlayson, 1978; Sigmundsson et al., 2010; Tibber et al., 2012; Anobile et al., 2013b), no study has been conducted on the relation between visual perceptual ability and symbolic numerical quantity comparison. In this study, we showed that visual perceptual ability was highly correlated with symbolic numerical quantity comparison, suggesting that visual form perception is critical for not only non-symbolic, but also symbolic numerical quantity comparison. As proposed in the Triple Code Model of Dehaene and Cohen (1995, 1997), visual code is about the representation of Arabic number forms. On the other hand, visual processing takes place in the occipito-temporal regions along the visual ventral processing path (e.g., Abboud et al., 2015). Thus, the close relation between visual form perception and symbolic numerical quantity comparison might be due to visual processing of Arabic number forms.

Moreover, the ANS is supra modal and can work with information from several modalities. Thus, there is a ratio-dependent performance in visual, auditory and haptic modalities (e.g., Barth et al., 2005, 2008; Jordan and Brannon, 2006; Jordan and Baker, 2011; Sasanguie et al., 2015; Gimbert et al., 2016). To our knowledge, no direct evidence has been found to show the link between auditory or haptic ANS acuity and mathematical performance, for which further empirical evidence is expected.

### Dissociated roles of visual form perception in exact and approximate computation

Among all six measures of general cognitive processes, visual form perception showed the highest correlation with exact computation, which is consistent with previous studies showing a close relationship between visual perception and exact computation (Halberda et al., 2008). Hierarchical regression analyses showed that the correlation between visual form perception and exact computation could not be fully accounted for by the other five general cognitive processes, suggesting that the cognitive component shared by visual form perception and exact computation is not shared by the other cognitive processes. The shared component may be number form processing, given that rapid processing of forms was involved in both visual form perception (e.g., figures) and exact computation (e.g., Arabic digits).

In contrast to its unique contribution to exact computation, visual form perception did not have a unique contribution to approximate computation after the other general cognitive processes were controlled for. Previous studies have shown a dissociation between exact and approximate computation in the brain (e.g., Dehaene et al., 1999). Exact computation is associated with brain regions involved in language processing, whereas approximate computation is associated with brain regions involved in spatial or quantity processing. It is possible that approximate computation recruits cognitive processes involved in quantity processing instead of those involved in number form processing, which might explain the absence of correlation between approximate computation and visual form perception. Future studies are needed to directly investigate why visual form perception shows a unique contribution to exact computation, but not to approximate computation.

The mediation analyses suggested that visual perception could be considered as a potential mechanism for the relationship between ANS and arithmetic. The positive effect from ANS training (e.g., Park and Brannon, 2013, 2014; Hyde et al., 2014) might originate from the enhancement of visual perception, that is, speed enhancement of visual perception might associate with arithmetic improvement. It is consistent with the viewpoint from Lindskog and Winman (2016) who suggested that the approximate arithmetic training promoted non-specific response speed. The non-specific response speed might be related with the visual perception speed.

### Visual form perception in mathematical achievement

Visual form perception did not show any significant contribution to curriculum-based mathematical achievement after controlling for gender, age and the other five general cognitive processes. However, exact computation, which was related with visual form perception, had a significant contribution to mathematical achievement. The contribution could be typically associated with some computational abilities beyond visual form perception, such as memory of computational facts and the application of computational procedures.

As mentioned earlier, the achievement test was curriculum-based and covered computation (including equations), mathematical concepts, and applied problem solving. The curriculum-based computation is the computation based on rational numbers, such as, |-$\sqrt{2}$|+ (cos60°- tan30°)°+ $\sqrt{8}$, x^4^÷x^5^y, $\frac{x}{2}$ + $\frac{x}{3}$ = $\frac{1}{4}$ (*x* + 3), which is beyond the scope of the exact computation task used in the current study. In this study, the students just leant the mathematics knowledge in the last semester, and thus they might not have acquired enough proficiency to solve the computational problems through simple visual inspection of symbols without significant mathematical processing. Instead, the students might have to use the majority of time and mental resources to search paths from the problem preconditions to the problem solution. The search process involves processing mathematical concepts and rules, which are centered on the semantic processing of mathematical knowledge and do not rely heavily on visual form perception.

Given that mathematical achievement tests cover a wide range of topics in the math curriculum, future studies should directly test the role of visual form perception in curriculum-based computation, concepts and mathematical problem solving.

Among all the general cognitive measures available in the current study, only non-verbal matrix reasoning had unique contribution to math achievement. Non-verbal matrix reasoning might be involved in the path search during the mathematical problem solving, which is a critical step of solving the mathematical achievement test. In contrast, visual processing was most likely involved in the initial perception and decoding of mathematical symbols, which is of less importance to the mathematical achievement test than path search.

### The roles of visual form perception in mathematical performance in development

Combining the current study and our previous studies (Zhou and Cheng, 2015; Zhou et al., 2015), we can conclude that the visual form perception could contribute to lower level math categories for students in primary and middle schools. Future study is needed to investigate the role of the visual form perception for younger children (e.g., in kindergarten) and for adults. According to the relation between visual form perception and exact computation, adults might have a significant association, but younger children might not have a significant association. The speculation could have some empirical evidence from the studies on developmental dyscalculia (DD).

Noël and Rousselle (2011) summarized existing studies and found that DD typically had poorer performance only in symbolic numerical processing for under 10 year old's, and had poorer performance in both non-symbolic (ANS) and symbolic numerical processing for above 10 years old's, compared to typically developing children (Rousselle and Noël, 2007; Iuculano et al., 2008; Landerl and Kölle, 2009; Landerl et al., 2009; Mussolin et al., 2010; Piazza et al., 2010; de Smedt and Gilmore, 2011; Mazzocco et al., 2011). Based on these results, Noël and Rousselle (2011) proposed that deficits in symbolic processing is directly related to mathematical difficulties, and that the association between the ANS and mathematical abilities is due to the influence of symbolic number processing in the ANS. A large-sample study on 626 5–7-year-old unselected children from the UK, China, Russia, and Kyrgyzstan found that it was the understanding of symbolic numbers that explained variations in mathematical performance in all samples, but not the ANS (Rodic et al., 2015). This view is consistent with our results that visual form processing is important for low-level math abilities, given that symbolic processing is closely related to visual form processing.

Thus, it appears that the relation between visual form processing and math abilities varies with age. More research is needed to assess this issue.

### Culture-related difference in math learning

Generally, math in China is taught in a similar way to that in Western countries. For example, Arabic digits are taught and used in math textbooks. The main difference may be that Chinese students typically spend more time on math learning (Stevenson et al., 1986). In addition, Chinese characters are used in math textbooks to explain math knowledge. The Chinese language belongs to a logographic system, different from alphabetic languages. Orthographic awareness or general visual perceptual skills are critical in the development of Chinese language abilities (e.g., Meng et al., 2002; Kuo et al., 2014). Chinese students' experience in acquisition and application of Chinese characters might transfer to the development of math knowledge, which might lead to greater reliance on visual form perception. To our knowledge, no study has directly compared degrees of reliance on visual form perception in mathematics for Chinese and Westerners.

Notably, visual form perception could be also important for participants in Western countries. Studies have shown a close relation between processing speed and mathematical performance for children in Western counties (e.g., Hitch and McAuley, 1991; Bull and Johnston, 1997; Fuchs et al., 2005). Processing speed was typically measured with tasks similar to our figure matching task. For example, Bull and Johnston (1997) used visual number matching task and geometric figure cross-out task (Kail and Hall, 1994), which were initially devised to assess the processing speed factor in the theory of fluid and crystallized intelligence (Cattell, 1963). Fuchs et al. (2005, 2008) also used similar tasks. Thus, the results in the current study may be applied to students speaking alphabetic languages, although future research is needed to directly compare the importance of visual form perception on mathematical performance between different cultural groups.

## Conclusions

Although the relation between visuospatial processing, especially visuospatial working memory and mathematics has been established, little research has been conducted to explore the roles of visual form perception as the starting point of visuospatial processing in mathematical performance. The current study investigated the relationships between visual form perception and five types of mathematical performance, covering non-symbolic and symbolic numerical quantity comparison, exact and approximate computation, and curriculum-based mathematical achievement. Visual form perception had independent contributions to numerosity comparison, digit comparison, exact computation, but no significant role on approximate computation and mathematics achievement in the final examination, after other general cognitive processes were controlled for. These results suggest that visual form perception may be an important cognitive basis for lower level math categories, including the approximate number system, digit comparison, and exact computation.

## Author contributions

JC and XZ designed research; YZ and DC performed research; JC and YZ analyzed data; and JC, DL, and XZ wrote the paper.

### Conflict of interest statement

The authors declare that the research was conducted in the absence of any commercial or financial relationships that could be construed as a potential conflict of interest.
